# Cost burden associated with advanced non-small cell lung cancer in Europe and influence of disease stage

**DOI:** 10.1186/s12885-019-5428-4

**Published:** 2019-03-08

**Authors:** Robert Wood, Gavin Taylor-Stokes

**Affiliations:** Adelphi Real World, Adelphi Mill, Bollington, Cheshire, SK10 5JB UK

**Keywords:** Non-small cell lung cancer, Disease stage, Work and productivity, Costs, Out-of-pocket expenses

## Abstract

**Background:**

Although evidence suggests that stage of disease may influence costs associated with non-small cell lung cancer (NSCLC), there remains a relative paucity of data on the financial burden incurred directly by patients and their informal caregivers as they progress through the disease course. As part of a large, cross-sectional study of the “real-world” humanistic and financial burden of advanced NSCLC in Europe, an analysis was conducted to quantify the cost burden of disease from a patient and caregiver perspective, and to evaluate how stage of disease impacts these costs.

**Methods:**

Financial data were collected (May 2015–June 2016) during a multinational (France, Germany, and Italy) cross-sectional study of adults with advanced NSCLC (stage IIIB–IV) and their informal (unpaid) caregivers. Data were obtained via medical chart reviews and patient/caregiver self-completion forms. Costs were annualized and unadjusted or adjusted for government financial support. Statistical significance was assessed using Mann-Whitney U tests.

**Results:**

One thousand thirty patients and 427 accompanying caregivers were recruited and asked to provide cost data. Mean total unadjusted direct and indirect out-of-pocket expenses were €5691 for patients and €4125 for caregivers; after adjusting for government financial support, values were €2644 and €3477. Mean wage losses were significantly higher for patients with stage IV vs IIIB NSCLC (€2282 vs €499; *p* = 0.0135) as were unadjusted direct out-of-pocket expenses (€4020 vs €1546; *p* = 0.0306). For caregivers, a similar but non-significant trend was observed. Mean total unadjusted direct and indirect out-of-pocket costs were numerically higher for stage IV vs IIIB NSCLC among patients (€5925 vs €3528) and caregivers (€4319 vs €2232); government financial support normalized patient costs, but they remained numerically higher for stage IV disease among caregivers.

**Conclusions:**

The financial burden of advanced NSCLC is considerable and appears to be influenced by stage of disease, with direct and indirect costs increasing as the disease progresses. Government financial support programmes appear to mitigate additional cost burdens among patients, but not among caregivers.

## Background

Incidence rates of lung cancer in some European countries are among the highest globally [1]. Across Europe, an estimated 400,000 new cases of lung cancer are diagnosed each year [1], and most cases, at least 85%, are non-small cell lung cancer (NSCLC) [2]. With the high mortality burden associated with the disease (an estimated 353,000 deaths each year), lung cancer represents a leading cause of cancer-related death in Europe [1, 3, 4].

The overall economic impact of lung cancer in Europe is substantial, with direct costs of caring for patients with the disease (including primary care, hospital inpatient/outpatient care, and drugs/oxygen) amounting to more than €3 billion per year [5]. When costs related to disability and premature mortality are considered as well, total annual costs amount to more than €100 billion [5]. While highlighting the significant societal cost of lung cancer, these cost estimates tend not to recognize the relatively high financial burden directly experienced by the patients and their caregivers as a result of the symptoms of the disease and associated psychological distress [6–9], the impact of cancer treatment and related side effects, and the effect of caregiving on the health and psychological wellbeing of the caregiver [10, 11].

There is a relative paucity of data on the cost burden incurred directly by patients with lung cancer and their informal caregivers in Europe. In a study of 128 patients with lung cancer receiving treatment at a University pulmonary department in Greece in 2011, and followed for 32 months, patients lost a total of 27,050 days of productivity and their caregivers lost a total of 1337 days of productivity [12]. Although no monetary value was assigned to this productivity loss, it would undoubtedly impact the earning potential of both patient and caregiver. In another study of 104 patients with NSCLC receiving treatment in 18 Italian oncology departments, costs related to principal (unpaid) caregiver support made up the vast majority (74%) of the overall assistance costs incurred per patient, highlighting the financial burden on informal caregivers [13].

Interestingly, the study by Gridelli et al. also suggested that the financial burden associated with caring for patients with NSCLC increases with the severity of lung cancer symptoms [13]. Furthermore, other studies have indicated that direct medical costs (physician and clinic visits, laboratory tests, medication charges) per patient with NSCLC appear to increase as the disease progresses [14, 15]. This evidence suggests that stage of disease may influence costs incurred by both patients with NSCLC and their caregivers.

Against this backdrop, as part of a large, cross-sectional study of the “real-world” humanistic and financial burden of advanced NSCLC in Europe, an analysis was conducted to quantify the cost of illness from a patient and caregiver perspective, and to evaluate how the stage of disease impacts on wage losses, out-of-pocket expenses, and productivity of patients with advanced NSCLC and their caregivers.

## Methods

### Study design and participating patients and caregivers

Cross-sectional data were derived from a study of patients with advanced NSCLC and their caregivers conducted in France, Germany, and Italy. Data were collected between May 2015 and June 2016 via medical chart review and separate patient and caregiver questionnaires. The study protocol was approved by a centralized institutional review board. All data were fully anonymized, collated, aggregated, and coded to permit linkage between physician-reported data, patient-reported outcomes, and caregiver responses.

To participate in the study, patients were required to be aged ≥18 years and have histologically or cytologically confirmed NSCLC with a diagnosis of locally advanced (stage IIIB) or metastatic (stage IV) disease. They also had to have initiated their first line of therapy for NSCLC at least 1 calendar month prior to data collection and had to be willing and able to complete patient questionnaires. Patients were not eligible for inclusion if they were participating in any clinical trial. Consecutive patients, who met the eligibility criteria, attending for consultation with a participating physician were invited to take part in the study. When patients were accompanied by their caregiver, the caregiver was also invited to participate in the study if they met the relevant eligibility criteria, as follows: an adult (aged ≥18 years) primary caregiver (spouse, partner, child, other relative, or friend) providing informal (unpaid) care for the patient. Participation for both patients and caregivers was entirely voluntary, and patients and caregivers were free to withdraw at any time without giving a reason.

### Data collection

Data collection consisted of three separate but linked components: a medical chart review, a patient self-completion questionnaire, and, where applicable, a caregiver self-completion questionnaire.

Physicians reviewed the medical charts of participating patients. Data were captured electronically and included, but were not limited to, patient demographics, diagnosis history, treatment history, and clinical characteristics. The self-completion components of the study included the collection of information on the patient’s and caregiver’s employment status, work productivity, and out-of-pocket expenses related to advanced NSCLC. Data from the patient and caregiver questionnaires were used to derive the relevant annual indirect and direct out-of-pocket costs associated with advanced NSCLC.

#### Cost data

Details on the derivation of cost data and associated cost calculations are provided in Table 1. Direct out-of-pocket expenses were defined as wage losses (per week); non-medical expenses associated with general practitioner or hospital visits (in the last 3 months); costs of treatments for conditions linked to NSCLC (in the last week), such as those for pain or symptom relief; and other non-medical costs arising from the diagnosis (per week), including additional childcare costs, assistance at home (cleaner, housekeeper, gardener), and travel costs. Indirect out-of-pocket costs were defined as productivity losses due to hours of work missed (absenteeism) and impaired productivity while at work (presenteeism). Work productivity and impairment at work data were derived using the Work Productivity and Activity Impairment Questionnaire: General Health (WPAI:GH) [16]. The WPAI:GH was included as one of the questionnaires completed by both patients and caregivers. The WPAI:GH consists of six items covering employment status, hours missed from work due to health problems, hours missed from work due to other reasons, hours actually worked, and a further two questions that measure the extent to which health problems affected productivity while working, and the ability to do regular activities. To determine the cost of absenteeism, hours missed from work due to NSCLC-related health problems in the last week obtained using the WPAI:GH were multiplied by the average hourly earnings per country. The cost of presenteeism was derived by multiplying the reported percentage impairment while working by the number of hours worked in the last week, with costings derived by multiplying the calculated number of impaired hours by the average hourly earnings per country. Cost calculations were adjusted to account for additional government financial support received by the patient or caregiver per week such as disability allowance, home care benefits, or sick leave remuneration. As cost data were collected based on short time horizons to reduce the likelihood of recall bias, annualized costs were derived for each cost component as described in Table 1.

**Table 1:** Derivation of cost data and associated cost calculations

Cost	Definition
Direct out-of-pocket costs^a^	
Wage losses (A)^b^	The reduction in the number of hours worked per week^c^ multiplied by the average hourly earnings in each country^d^. Annualized by multiplying by 48^e^.
Non-medical GP/hospital visit expenses (B)	The reported number of visits to the GP/hospital in the last 3 months was annualized by multiplying by 4. This product was then multiplied by the sum of transportation costs to GP/hospital and other out-of-pocket expenses when visiting the GP/hospital.
Secondary NSCLC-related treatment costs (C)^f^	The amount paid in the last week for treatments for conditions linked to NSCLC (e.g., pain or other symptom relief). Annualized by multiplying by 52.
Other non-medical monetary burden costs (D)	Cost of additional non-medical resources required per week as a result of NSCLC (e.g., child care, housekeeper/cleaner, gardener, taxi/transportation). Annualized by multiplying by 52.
Indirect out-of-pocket costs^g^	
Productivity losses (E)^h^	Sum of cost of absenteeism (hours of work missed in the last week^i^ due to NSCLC-related health problem multiplied by the average hourly earnings per country^d^) and cost of presenteeism (percentage impaired while working^i^ multiplied by the number of hours worked in the last 7 days, multiplied by the average hourly earnings per country^d^). Annualized by multiplying by 48^e^.
Government financial support	
Government financial support (F)	Financial support received by the patient or caregiver from the government per week (e.g., disability allowance, home care benefits, sick leave remuneration). Annualized by multiplying by 52.
Cost calculation	Formula
Direct out-of-pocket expenses (unadjusted)	A + B + C + D
Direct out-of-pocket expenses (adjusted)^j^	(A + B + C + D) - F
Total direct and indirect out-of-pocket costs (unadjusted)^k^	A + B + C + D + E
Total direct and indirect out-of-pocket costs (adjusted)^j^	(A + B + C + D + E) - F

*NSCLC* non-small cell lung cancer

^a^NSCLC costs directly borne by the patient or caregiver

^b^Annual wage loss was assumed to be €0.00 for patients/caregivers who were not in employment prior to NSCLC diagnosis/caring for the patient with NSCLC

^c^If no change in employment status, this was assumed to be 0 h; if changed to unemployed, assumed average hours per working week in each country, based on Eurostat 2015 estimates – France: 37.2 h; Germany: 35.2 h; Italy: 37.0 h

^d^Based on Eurostat 2014 estimates – France: €17.40; Germany: €17.78; Italy: €15.54

^e^Assumes 48 working weeks per year

^f^Costs for primary NSCLC treatment (e.g., chemotherapy) are not included; only includes costs related to secondary medications required to address NSCLC-related symptoms

^g^NSCLC costs not borne by patients or caregivers (i.e., cost to employers)

^h^Productivity losses for patients and caregivers not in current employment were conservatively assumed to be €0.00

^i^Measured using the Work Productivity and Activity Impairment Questionnaire: General Health [16]

^j^Adjusted for financial support provided by the respective governments

^k^Represents the sum of all cost components, but does not account for government financial support subsidizing patient or caregiver direct costs

### Statistical analysis

Descriptive statistics are presented throughout. Outcomes are presented for the total population with available cost data or stratified by stage of NSCLC (stage IIIB vs stage IV). Cost data were not complete for all participants as they were free to omit responses at their own choice. Relevant sample sizes are reported. Missing data were not imputed and thus remain missing. Statistical significance was assessed using Mann-Whitney U tests. All statistical tests performed were two-sided in nature and a significance level of 0.05 was used. All analyses were performed using Stata software (StataCorp. 2015. Stata Statistical Software: Release 14 or later. College Station, TX, USA: StataCorp LLC).

## Results

### Patient population

Cost data were derived for 1030 patients (France, *n* = 351; Germany, *n* = 347; Italy, *n* = 332) and 427 accompanying informal caregivers (France, *n* = 148; Germany, *n* = 150; Italy, *n* = 129) across the three participating European countries. Demographics and clinical characteristics for the patients are shown in Table 2. Overall, 119 patients (11.6%) had stage IIIB and 911 (88.4%) had stage IV disease. Patients with stage IV disease were slightly older (mean age: 64.8 vs 62.4 years), had a longer disease duration (mean: 37.1 vs 27.6 weeks), and were more likely to receive second- or later-line therapy (31.1% vs 17.6%) than patients with stage IIIB disease. Rates of comorbidities were generally comparable, although hypertension was more common (34.1% vs 21.2%) and anxiety less common (14.6% vs 21.2%) in those with stage IV disease. Only a minority of patients were in full- or part-time employment at the time of the study, regardless of stage of disease; this proportion was lower among patients with stage IV compared with stage IIIB disease (20.3% vs 27.1%).

**Table 2:** Patient demographics and clinical characteristics

	Overall (*N* = 1030)	Stage IIIB (*n* = 119)	Stage IV (*n* = 911)
Mean age^a^, years (SD)	64.5 (10.1)	62.4 (10.6)	64.8 (10.0)
Male sex, *n* (%)	679 (65.9)	86 (72.3)	593 (65.1)
Mean body mass index^b^, kg/m^2^ (SD)	24.0 (3.4)	24.4 (3.7)	24.0 (3.3)
Current/former smoker^c^, *n* (%)	787 (77.9)	98 (83.8)	689 (77.2)
Histological tumour type, *n* (%)			
Non-squamous	724 (70.3)	73 (61.3)	651 (71.5)
Squamous	306 (29.7)	46 (38.7)	260 (28.5)
Mean disease duration^d^, weeks (SD)	35.9 (45.0)	27.6 (40.1)	37.1 (45.5)
Receiving second- or later-line therapy^e^, *n* (%)	302 (29.5)	21 (17.6)	281 (31.1)
ECOG PS, *n* (%)			
0	200 (19.4)	24 (20.2)	176 (19.3)
1	448 (43.5)	53 (44.5)	395 (43.4)
2	282 (27.4)	29 (24.4)	253 (27.8)
3	82 (8.0)	13 (10.9)	69 (7.6)
4	18 (1.7)	0 (0.0)	18 (2.0)
Comorbidities^f^, *n* (%)			
COPD	362 (35.3)	36 (30.5)	326 (35.9)
Hypertension	335 (32.7)	25 (21.2)	310 (34.1)
Anxiety	158 (15.4)	25 (21.2)	133 (14.6)
Hyperlipidaemia	131 (12.8)	16 (13.6)	115 (12.7)
Depression	112 (10.9)	15 (12.7)	97 (10.7)
Diabetes mellitus	112 (10.9)	15 (12.7)	97 (10.7)
Emphysema	107 (10.4)	8 (6.8)	99 (10.9)
Ischaemic heart disease	90 (8.8)	7 (5.9)	83 (9.1)
Bronchitis	87 (8.5)	8 (6.8)	79 (8.7)
Peripheral vascular disease	67 (6.5)	4 (3.4)	63 (6.9)
None	314 (30.6)	35 (29.7)	279 (30.7)
Current employment status^g^, *n* (%)			
Retired	614 (60.4)	66 (55.9)	548 (61.0)
Working (full-time)	149 (14.7)	28 (23.7)	121 (13.5)
Working (part-time)	65 (6.4)	4 (3.4)	61 (6.8)
Unemployed	105 (10.3)	11 (9.3)	94 (10.5)
Other^h^	83 (8.2)	9 (7.6)	74 (8.2)

*COPD* chronic obstructive pulmonary disease; *ECOG PS* Eastern Cooperative Oncology Group Performance Status; *NSCLC* non-small cell lung cancer; *SD* standard deviation

Patient numbers given at top of column apply in all cases unless noted below

^a^Patients reported to be 90+ years of age were assumed to be 90 years of age for the purposes of this analysis; overall, *N* = 1028; stage IV, *n* = 909

^b^Overall, *N* = 926; stage IIIB, *n* = 108; stage IV, *n* = 818

^c^Overall, *N* = 1010; stage IIIB, *n* = 117; stage IV, *n* = 893

^d^Overall, *N* = 1015; stage IV, *n* = 896

^e^Overall, *N* = 1022; stage IV, *n* = 903

^f^Comorbidities shown are those occurring in more than 5% of patients in either NSCLC stage subgroup; overall, *N* = 1026; stage IIIB, *n* = 118; stage IV, *n* = 908

^g^Overall, *N* = 1016; stage IIIB, *n* = 118; stage IV, *n* = 898

^h^Includes homemakers and students

Regardless of line of therapy, most enrolled patients receiving treatment were receiving a chemotherapy-based regimen (76.2%). The type of regimen differed depending on the line of therapy, with chemotherapy doublets or triplets (with or without a targeted agent) more common as a first-line therapy than as a second- or later-line therapy (67.1% vs 16.9%) and single-agent chemotherapy (with or without a targeted agent) more common as a second- or later-line therapy than a first-line therapy (49.7% vs 13.2%).

Characteristics of the accompanying informal caregivers are shown in Table 3. Thirty (7.0%) were caring for a patient with stage IIIB NSCLC and 397 (93.0%) were caring for a patient with stage IV disease. Caregivers were principally family members, with partners/spouses (54.9%) and children (31.9%) making up the majority. This pattern of caregiver–patient relationship was consistent across the stage IIIB and stage IV groups. Fewer than half (45.0%) of caregivers were in full- or part-time employment at the time of the study, although data were not collected on whether the unemployed caregivers had previously been working or had never worked. Employment status was generally comparable between the stage IIIB and stage IV groups, although a noticeably greater proportion of caregivers for patients with stage IV than caregivers for patients with stage IIIB disease were in full-time employment (37.5% vs 17.9%, respectively).

**Table 3:** Caregiver demographics

	Overall (*N* = 427)	Stage IIIB (*n* = 30)	Stage IV (*n* = 397)
Mean age^a^, years (SD)	53.5 (12.5)	54.7 (12.1)	53.4 (12.5)
Male sex^b^, *n* (%)	116 (27.4)	6 (20.7)	110 (27.9)
Relationship to patient^c^, *n* (%)			
Partner/Spouse	234 (54.9)	16 (53.3)	218 (55.1)
Daughter/Son	136 (31.9)	8 (26.7)	128 (32.3)
Sister/Brother	11 (2.6)	0 (0.0)	11 (2.8)
Mother/Father	3 (0.7)	0 (0.0)	3 (0.8)
Other family member	12 (2.8)	2 (6.7)	10 (2.5)
Friend/Neighbour	13 (3.1)	0 (0.0)	13 (3.3)
Other	3 (0.7)	2 (6.7)	1 (0.3)
None	14 (3.3)	2 (6.7)	12 (3.0)
Current employment status^d^, *n* (%)			
Working (full-time)	152 (36.2)	5 (17.9)	147 (37.5)
Working (part-time)	37 (8.8)	6 (21.4)	31 (7.9)
Retired	106 (25.2)	6 (21.4)	100 (25.5)
Unemployed	35 (8.3)	2 (7.1)	33 (8.4)
Other^e^	90 (21.4)	9 (32.1)	81 (20.7)

*SD* standard deviation

Patient numbers given at top of column apply in all cases unless noted below

^a^Overall, *N* = 425; stage IV, *n* = 395

^b^Overall, *N* = 423; stage IIIB, *n* = 29; stage IV, *n* = 394

^c^Overall, *N* = 426; stage IV, *n* = 396

^d^Overall, *N* = 420; stage IIIB, *n* = 28; stage IV, *n* = 392

^e^Includes homemakers and students

### NSCLC cost analyses – overall populations

Patient and caregiver costs related to advanced NSCLC for the overall study populations are shown in Table 4. There were 155 instances for which the unadjusted total direct and indirect out-of-pocket costs could be calculated for both the patient with advanced NSCLC and their accompanying caregiver; the mean total annual costs for this subgroup were €9202 per patient–caregiver dyad.

**Table 4:** Costs related to advanced NSCLC stratified by patients and caregivers

Mean annual costs per patient/caregiver	Patients (*N* = 1030)	Caregivers (*N* = 427)
Wage losses	*n* = 1000 €2077	*n* = 360 €436
Direct out-of-pocket expenses (unadjusted)	*n* = 544 €3774	*n* = 213 €1756
Direct out-of-pocket expenses (adjusted^a^)	*n* = 539 €823	*n* = 203 €1019
Productivity losses	*n* = 966 €1484	*n* = 387 €2839
Total direct and indirect out-of-pocket costs (unadjusted)	*n* = 522 €5691	*n* = 204 €4125
Financial support received from government	*n* = 989 €2110	*n* = 386 €721
Total direct and indirect out-of-pocket costs (adjusted^a^)	*n* = 518 €2644	*n* = 194 €3477

*NSCLC* non-small cell lung cancer

^a^Adjusted for financial support provided by the respective governments

### NSCLC cost analyses – stage IIIB vs stage IV disease

**Fig. 1:**
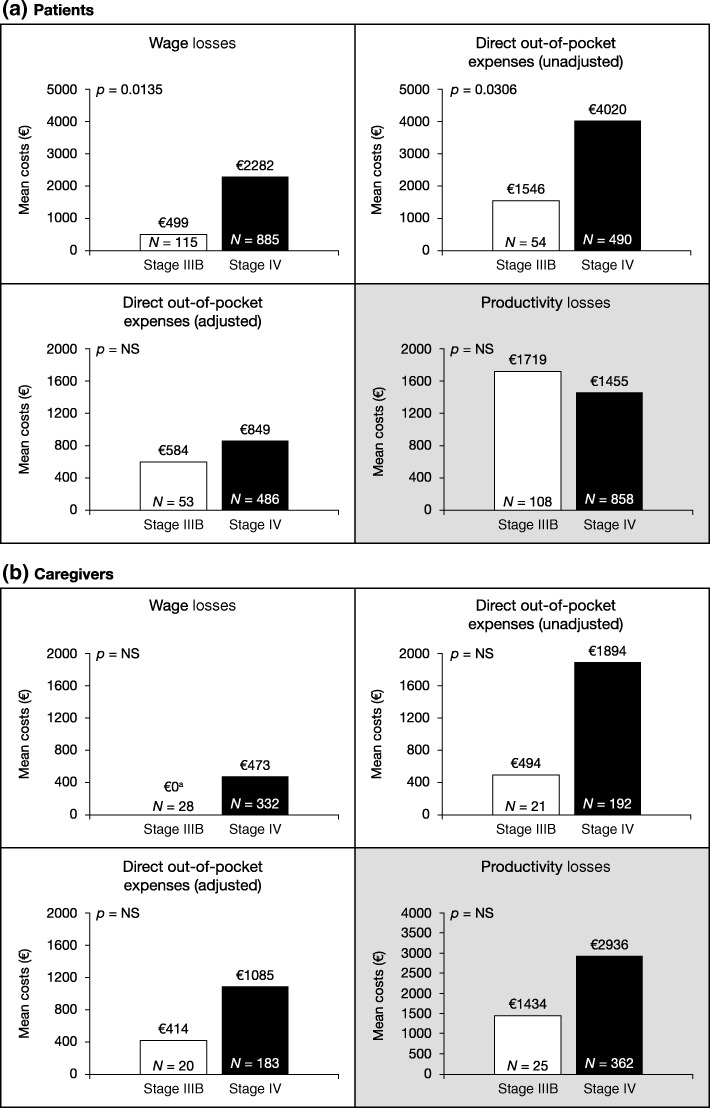
Direct and indirect out-of-pocket costs by stage of disease for patients (**a**) and caregivers (**b**). *NS* not significant. White boxes are costs directly borne by the patient/caregiver; grey boxes represent indirect costs borne by employers. ^a^ Indicates that no caregiver’s employment status was impacted by caring for a patient

Patients with stage IV disease had significantly higher wage losses, and incurred significantly higher direct out-of-pocket expenses, than those with stage IIIB disease when government financial support was not considered. Indirect out-of-pocket costs (productivity losses) were, in contrast, similar for patients with stage IIIB and stage IV disease (Fig. 1a). Overall, the total unadjusted direct and indirect out-of-pocket costs were substantially higher for patients with stage IV vs stage IIIB disease (Table 5). However, financial support from the government was also significantly higher for patients with stage IV vs stage IIIB disease (mean annual value of €2282 vs €737; *p* = 0.0083; Table 5). As such, when government financial support was considered, both the adjusted direct out-of-pocket expenses (Fig. 1a) and adjusted total direct and indirect out-of-pocket costs (Table 5) were relatively similar for patients with stage IIIB and stage IV disease, with no statistically significant difference between the two patient groups.

**Table 5:** Total direct and indirect out-of-pocket costs accrued by patients with advanced NSCLC and their caregivers by disease stage

	Patients		Caregivers	
Mean annual cost outcome	Stage IIIB (*n* = 119)	Stage IV (*n* = 911)	Stage IIIB (*n* = 30)	Stage IV (*n* = 397)
Total direct and indirect out-of-pocket costs (unadjusted)	*n* = 51 €3528	*n* = 471 €5925	*n* = 19 €2232	*n* = 185 €4319
Financial support received from government	*n* = 110 €737	*n* = 879 €2282	*n* = 29 €663	*n* = 357 €726
Total direct and indirect out-of-pocket costs (adjusted^a^)	*n* = 50 €2548	*n* = 468 €2654	*n* = 18 €2241	*n* = 176 €3603

*NSCLC* non-small cell lung cancer

^a^Adjusted for financial support provided by the respective governments

For caregivers, although differences were not statistically significant, both wage losses and unadjusted direct out-of-pocket expenses were numerically higher (at least double) among caregivers of patients with stage IV vs stage IIIB disease. Caregiver productivity losses were also substantially higher among caregivers of patients with stage IV disease (Fig. 1b). Regardless of the patient’s stage of disease, the amount of financial support from the government for caregivers was relatively consistent (mean annual value of €663 for stage IIIB and €726 for stage IV). Consequently, when financial support from the government was taken into consideration, the adjusted direct out-of-pocket expenses (Fig. 1b) and adjusted total direct and indirect out-of-pocket costs (Table 5) remained noticeably higher for those caring for patients with stage IV disease than for those caring for patients with stage IIIB disease.

## Discussion

The societal cost burden associated with advanced NSCLC is considerable. In Europe, costs including primary care physician visits, hospital inpatient/outpatient visits, medication, and supportive therapies such as oxygen, amount to more than €3 billion per year [5]. Less well defined are the out-of-pocket expenses accrued directly by the patients themselves and their caregivers. The current analysis was undertaken to better define this cost burden and has revealed mean annual out-of-pocket expenses of €3774 per patient and €1756 per caregiver. These direct out-of-pocket expenses relate to lost wages; costs accrued in relation to physician visits; costs associated with supportive medications for symptom relief or pain management; and other costs related to additional NSCLC-related factors, such as child care, housekeeping/cleaning, and transportation. Moreover, when indirect costs related to productivity losses were included, the combined total direct and indirect out-of-pocket costs exceeded €9000 per patient–caregiver dyad. In calculating the out-of-pocket cost burden of advanced NSCLC among the participating patients and caregivers, financial remuneration received through governmental assistance systems was also considered. Such assistance schemes varied between countries but generally included disability allowance payments, home care benefits, and sick leave remuneration. When combined for patients and caregivers (in a subset of 144 patient–caregiver dyads with full data), the government support amounted to around one-half of the out-of-pocket costs incurred by patients and their caregivers (mean annual support, €4782; mean annual total unadjusted direct and indirect out-of-pocket costs, €9634).

The population participating in this study included patients with locally-advanced (stage IIIB) or metastatic (stage IV) NSCLC. Current data suggest that around 50–60% of patients with NSCLC are diagnosed at these advanced stages, when 5-year survival rates are below 5% [17–19]. In the analysis of costs by disease stage, patients with stage IV NSCLC incurred significantly greater wage losses (*p* = 0.0135) and unadjusted direct out-of-pocket expenses (*p* = 0.0306) than those with stage IIIB disease. The statistical significance between the stages was lost when the direct out-of-pocket expenses were adjusted for governmental financial support received by patients. Indeed, the bulk of the additional out-of-pocket expenses incurred by patients with stage IV disease were offset by additional government financial support, although mean costs remained numerically higher for the patients with stage IV disease.

For caregivers, wage losses and direct out-of-pocket expenses were numerically higher for those caring for patients with stage IV disease than those caring for patients with stage IIIB disease although the differences, with or without adjustment for government financial support, did not reach statistical significance. Of note, the current study was conducted in European countries with established social and financial support systems; in countries with less well-established or comprehensive government financial support systems, such costs would presumably be borne by the patient and/or caregivers.

With regard to productivity losses, a cost borne by employers rather than the individual patient or caregiver, costs per patient did not appear to be majorly influenced by stage of disease. In contrast, the mean cost of productivity losses per caregiver of a patient with stage IV disease was more than double the mean cost per caregiver of a patient with stage IIIB disease, although the difference did not reach statistical significance. This observation may reflect the higher proportion of caregivers of patients with stage IV disease (vs caregivers of patients with stage IIIB disease) who were in full-time employment and the consequent greater scope for caregiving to disrupt their working life.

Previous reports have shown that direct medical costs per patient with NSCLC appear to increase with disease progresses. In 2008, Fox et al. reported the results of a retrospective analysis of cost data for 306 patients with stage IIIB or stage IV NSCLC who were receiving chemotherapy, and compared costs for those with stable disease vs those with progressive disease (defined as a change in chemotherapy regimen and radiologic confirmation of tumour growth) [14]. The total direct cost of care in the 3 months following disease progression increased by approximately one-third compared with the costs of care for patients with stable disease (USD $31,129 vs $18,802, respectively). More recently, Migliorino et al. found that costs including those related to physician visits, hospitalizations, medication, laboratory tests, and palliative care increased as patients experienced disease progression and received sequential lines of therapy [15]. A similar pattern of costs increasing with disease progression was also reported for patients with stage IIIB or stage IV disease receiving treatment with tyrosine kinase inhibitors, with direct medical and associated healthcare costs increasing with disease progression [20]. The current evaluation adds to these observations and has shown that direct out-of-pocket expenses incurred by the patient are influenced by the disease stage.

Prior observational studies have also demonstrated the cost burden of caring for patients with advanced NSCLC. In an analysis of data derived from the 2010/2011 EU National Health and Wellness Survey (covering relatives of patients with lung cancer in France, Germany, Italy, Spain, and the UK) relatives providing care for a patient with lung cancer reported significantly greater work impairments and were associated with higher indirect costs (productivity losses) compared with relatives not providing care [11]. The current analysis takes this further by showing the direct impact of advanced NSCLC on out-of-pocket expenses incurred by the caregiver. Moreover, contrary to the situation observed for the patients, the burden of caregiver expenses and costs was only partly compensated for by government financial support. Indeed, this support was considerably lower for caregivers of patients with advanced NSCLC than for the patients themselves. Consequently, the out-of-pocket expenses directly incurred by caregivers tended to be higher for those caring for patients with a more advanced stage of disease. Costs for caregivers of patients with NSCLC increasing in tandem with symptom severity has been reported previously, and may be regarded as a proxy for advancing disease [13]. Furthermore, Van Houtven et al. have previously shown that caregiver costs increased with increasing stage at diagnosis among carers of patients with lung or colorectal cancer [21]. In this cross-sectional study conducted in the US, caring for a patient diagnosed with lung or colorectal cancer at late stage (stage IV) was associated with a 53.9% higher economic burden than caring for a patient diagnosed at early stage (stage I; *p* = 0.001).

The current analysis differs from conventional “cost of illness” studies in that it did not consider the costs of primary treatments for NSCLC (i.e., costs of chemotherapy, etc.) and therefore allowed a more focused assessment of the financial burden on patients and their caregivers, as well as costs to the government and employers in relation to the patients and their caregivers. Although such analyses have previously been conducted for caregivers, there is limited literature on the influence of disease stage on direct out-of-pocket costs to patients, and this analysis has confirmed such costs to be substantial.

The strengths of the current analysis included the cross-sectional, multinational design and the multidimensional approach including wage losses, out-of-pocket expenses, and productivity losses. In addition, by recruiting consecutive patients attending for physician consultation, the potential for selection bias was limited. Moreover, by excluding patients actively involved in clinical trials at the time of data collection, the opportunity to capture “real-world” cost data was increased.

The reliance on participant self-reporting and, because the analysis was based on cross-sectional data rather than longitudinal data, the associated reliance on the recall of the participant, means that the cost data should be considered an over or underestimation of true costs. A further potential limitation was that, as participants could omit responses, cost data were missing for a proportion of patients and caregivers – the resultant relatively small sample size for some subgroups is likely the reason that statistical significance was rarely seen. Furthermore, although the questionnaires contained explicit instructions aimed at focusing the cost impact on the patient and caregiver separately, the possibility of some double costing cannot be discounted. Another potential limitation is the use of average country-specific earnings in the calculation of wage losses, which may not be optimal for this older population whose income may reflect a higher-earning, more experienced workforce. In addition, the study did not capture information on all factors that could influence cost burden on patients and caregivers. It must be acknowledged that a wide variety of factors could potentially influence the economic burden, particularly for caregivers (e.g., age of caregiver, presence of children at home, etc.) [22]. It is also important to recognize that, because this study almost exclusively included patients receiving systemic therapy (primarily chemotherapy), the absolute costs reported may be somewhat conservative. Based on recent large-scale real-world research, between one- and two-thirds of patients with advanced NSCLC do not receive systemic therapy [17, 19]. Since it would be fair to assume that the patients contraindicated for chemotherapy would be the sickest, it is possible that costs related to their care would surpass those for the patients evaluated in this study. Finally, it must be noted that the costs reported herein were captured based on relatively short time horizons (a week up to 3 months) and then annualized, but only a minority of patients with stage IIIB–IV disease survive for a year after diagnosis [17], further emphasizing that the cost data is likely an over-estimate of true costs.

## Conclusions

The current analysis supports others that suggest the financial burden of advanced NSCLC is considerable to society, patients, and their caregivers, and appears to be influenced by stage of disease, with direct and indirect costs increasing as the disease progresses. To our knowledge, this study is the first to demonstrate the influence of stage of disease on direct patient wage-related and out-of-pocket costs including the cost of secondary NSCLC–related treatments, non-medical costs related to primary care and hospital visits, and other non-medical monetary burden costs. Beyond this, the findings of these analyses also suggest that, while government financial support programmes may be somewhat successful in taking much of the increased cost burden away from patients as the disease advances to the metastatic stage, the same cannot be said for caregivers. The implications of the observations reported here are that there is a need for newer, more effective, and safer treatments for NSCLC that slow or even prevent disease progression. In addition, the results might have more wide-ranging implications such as (i) highlighting a need for improved financial support programmes aimed at easing the economic burden of caring for patients with advanced NSCLC, particularly in relation to non-spousal caregivers who may not benefit from patient-focused financial support; and (ii) suggesting that financial counselling/advice needs to be tailored for NSCLC patient–caregiver dyads as the disease advances to the metastatic stage.

## Abbreviations

COPD: Chronic obstructive pulmonary disease
ECOG PS: Eastern Cooperative Oncology Group Performance Status
NS: Not significant
NSCLC: Non-small cell lung cancer
SD: Standard deviation
WPAI:GH: Work Productivity and Activity Impairment Questionnaire: General Health
